# Comparison between planar and single-photon computed tomography images for radiation intensity quantification in iodine-131 scintigraphy

**DOI:** 10.1038/s41598-021-01432-x

**Published:** 2021-11-08

**Authors:** Yusuke Iizuka, Tomohiro Katagiri, Minoru Inoue, Kiyonao Nakamura, Takashi Mizowaki

**Affiliations:** Department of Radiation Oncology and Image-Applied Therapy, Graduate School of Medicine, Kyoto University, 54, Shogoin Kawahara-cho, Sakyo-ku, Kyoto-shi, Kyoto, 606-8507 Japan

**Keywords:** Medical research, Oncology

## Abstract

This study aimed to evaluate the feasibility of quantifying iodine-131 (^131^I) accumulation in scintigraphy images and compare planar and single-photon emission computed tomography (SPECT) images to estimate ^131^I radioactivity in patients receiving radioactive iodine therapy for thyroid cancer. We evaluated 72 sets of planar and SPECT images acquired between February 2017 and December 2018. Simultaneously, we placed a reference ^131^I capsule next to the patient during image acquisition. We evaluated the correlation between the intensity of the capsule in the images and the capsule dose and estimated the radiation dose at the thyroid bed. The mean capsule dose was 2.14 MBq (range, 0.63–4.31 MBq). The correlation coefficients (*p*-value) between capsule dose and maximum and mean intensities in both planar and SPECT images were 0.93 (*p* < 0.01), 0.96 (*p* < 0.01), 0.60 (*p* < 0.01), and 0.47 (*p* < 0.01), respectively. The mean intensities of planar images show the highest correlation coefficients. Based on a regression equation, the average radiation dose in the thyroid bed was 5.9 MBq. In conclusion, planar images reflected the radiation dose more accurately than SPECT images. The regression equation allows to determine the dose in other regions, such as the thyroid bed or sites of distant metastasis.

## Introduction

The most common cancer in the endocrine system is thyroid cancer^1^, and its most frequent type differentiated thyroid cancer (DTC), such as papillary and follicular carcinomas, being found in over 90% of cases.

After total thyroidectomy, radioactive iodine (RAI) therapy with iodine-131(^131^I) is a widely accepted treatment for DTC^2^. According to the guidelines, the aims of RAI therapy are divided into three categories^3^: ablation of the remnant thyroid tissue to simplify follow-up, adjuvant therapy for microscopic lesions to decrease the risk of recurrence or distant metastasis, and treatment for tangible residual or metastatic diseases.

Generally, the dose of the ^131^I drug is hardly defined based on the tumor or normal tissue; instead, the radioactivity of RAI drugs is empirically fixed. For example, 1,110 MBq is sufficient for remnant ablation, while adjuvant therapy and treatment require 3,700 to 7,400 MBq, according to guidelines^3^. Some reports administered ^131^I drug based on bone marrow or blood level dosimetry, but it is not a popular procedure^4^. Although there are some general models to simulate the kinetics of radioisotopes in humans, such as the medical internal radiation dose method^5^, there is no established method to evaluate the strength of tumor or normal tissue radiation in RAI therapy with ^131^I in single patients.

Planar imaging or single-photon emission computed tomography (SPECT) has been used for qualitative evaluation of radioisotope distributions, with some reports focusing on the quantitative analysis of several low-energy radioisotopes, such as ^99m^Tc, ^123^I, etc.^6–8^. These quantitative values are used to evaluate treatment response or predictive factors in the oncology field. In the field of RAI therapy, ^131^I distribution can be evaluated with scintigraphy acquired with a gamma camera a few days after ^131^I drug administration. However, in general, it is difficult to quantitatively evaluate ^131^I uptake because of the elevated energy of its gamma rays, collimator penetration, and artifacts, which cannot be disregarded in quantitative analysis. As a result, thus far, there has been no quantitative analysis or direct comparison between planar and SPECT images of ^131^I scintigraphy.

We hypothesized that we could quantitatively estimate ^131^I uptake based on a known radiation dose source, which is placed such that it is imaged by the same camera at the same time. In this study, we developed methods for the quantitative evaluation of ^131^I uptake in planar and SPECT images using scintigraphy. In addition, based on quantitative analysis, we estimated the radiation dose at the thyroid bed in patients with thyroid cancer who underwent RAI therapy.

## Results

### Comparison between the planar and SPECT images

**Figure 1:**
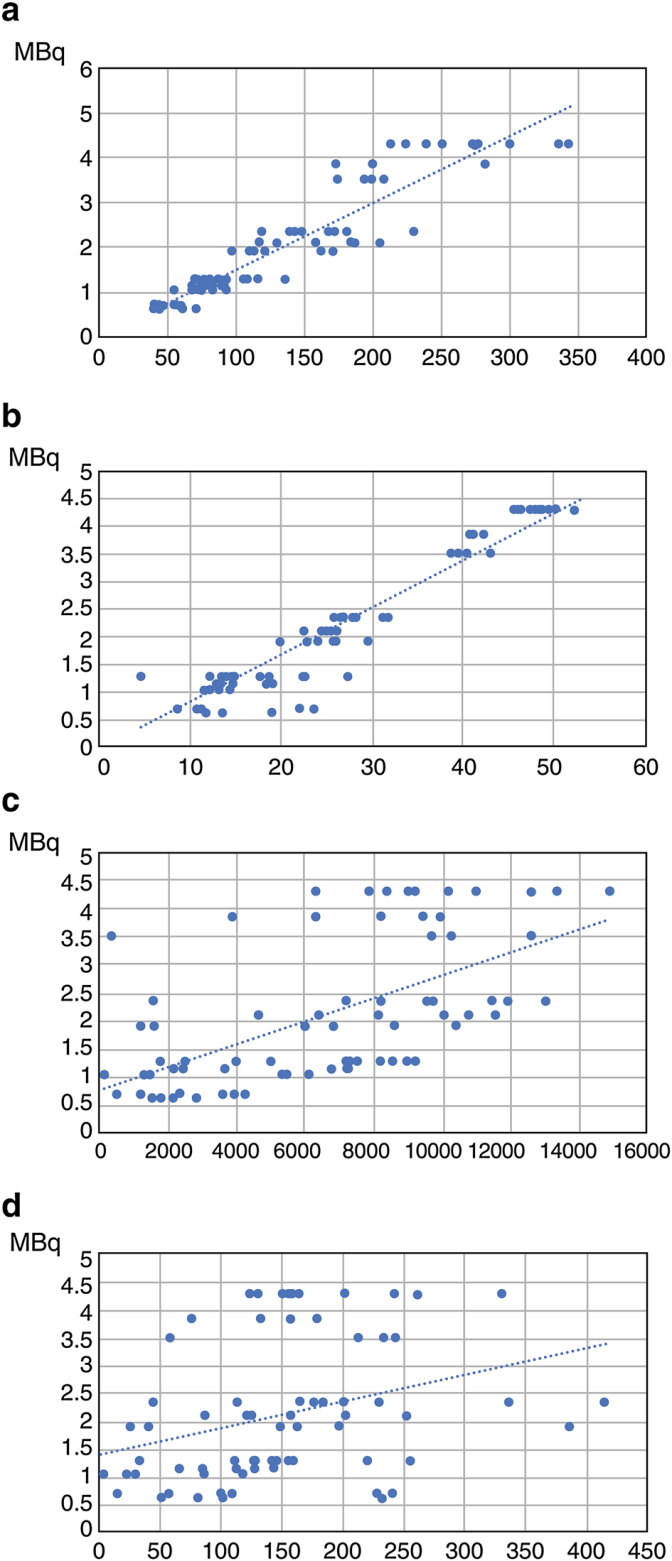
The scatter plots and regression lines of the relationship between the intensity and the actual radiation dose. The intensity is shown in maximum intensity in the planar image (**a**), averaged intensity in the planar image (**b**), maximum intensity in the single-photon emission computed tomography (SPECT) image (**c**), and averaged intensity in the SPECT image (**d**).

We successfully obtained ^131^I intensity data in all images using the treatment planning support system. The 37, 111, and 185 MBq of ^131^I capsules were used as references, and the exact doses of the capsules were calculated with the time between the test day and the image acquisition. The mean ^131^I capsule dose was 2.14 MBq (range, 0.63–4.31 MBq). The correlation coefficients (*p*-value) between the capsule doses and the maximum and mean intensities in planar images and maximum and mean intensities in SPECT images were 0.93 (*p* < 0.01), 0.96 (*p* < 0.01), 0.60 (*p* < 0.01), and 0.47 (*p* < 0.01), respectively (Fig. 1, Table 1). The mean intensities of planar images show the highest correlation coefficients. In post hoc power analysis, the statistical power was 0.98, when two-sided, the effective size was 0.5, and the alpha error 0.01. Based on the mean intensities in planar images, the regression equation can be expressed as y(dose) = 0.0845 × (mean intensity in the planar image).

**Table 1:** The relationship between the intensity and the actual radiation dose.

	Planar		SPECT	
	Maximum	Mean	Maximum	Mean
Correlation coefficients	0.93	0.96	0.60	0.47
*p*-value	< 0.01	< 0.01	< 0.01	< 0.01

*SPECT* single-photon emission computed tomography.

### Estimation of the dose at the thyroid bed

Among all patients, in the 47 who underwent RAI therapy for the first time, their uptake was observed at the thyroid bed. Based on the above regression equation, the average (range) radiation dose at the thyroid bed was 5.9 MBq (0.1–45.1 MBq).

## Discussion

In the present study, we were able to calculate radiation doses from scintigraphy images. By comparing planar and SPECT images, we determined the proper image to estimate the radiation dose based on scintigraphy, finding that planar images are appropriate for radiation dose estimation.

Recently, the importance of dosimetry has increased in nuclear medicine, especially in radioisotope therapy, because the absorbed dose of the tumor directly correlates with effectiveness. Although several studies have reported on quantitative dose estimations for radioisotopes, such as ^99m^Tc and ^123^I in scintigraphy, to the best of our knowledge, this is the first report to suggest the possibility of dose estimation in ^131^I scintigraphy.

There are some commercial tools for the quantitative analysis of bone scintigraphy. For example, the bone scan index in BONENAVI (Fujifilm RI Pharma Co. Ltd., Tokyo, Japan) with planar images and standardized uptake values in GI-BONE (AZE, Tokyo, Japan) with SPECT images have been well reported^9–11^. These quantitative indicators are used for the prediction or evaluation of therapeutic effects or predictive factors for survival in the oncology field^12,13^. With progress in this study and the accumulation of more data points, we could predict the therapeutic effect or survival rate in the treatment of DTC.

There have been several attempts to quantitatively evaluate ^131^I. For example, van Gils et al. suggested a method for estimating the absorbed thyroid dose with a collar detector and SPECT images. They assumed a two-compartment model fit and showed good agreement with the measured data points in three patients^14^. Minguez et al. analyzed SPECT/CT dosimetry with whole-remnant and maximum-voxel methods, and could estimate the absorbed dose at the thyroid bed in patients who underwent ablation^15^. However, the purpose of these reports was different from that of this study, and none aimed to directly compare planar and SPECT images in ^131^I scintigraphy. There has been no established dosimetry method of ^131^I accumulation based on the scintigraphy; our method based on this study may be one way to calculate the radiation dose.

Planar images reflected the radiation dose more accurately than SPECT images in this study, and according to the post hoc analysis, this result was reproducible and robust. We expected that SPECT images would show a better correlation between the actual dose and intensity because SPECT images are acquired using CT images with absorption compensation. In addition, the spatial resolution of SPECT images is better than that of planar images, calculating the absorbed dose with CT images, such as external beam radiotherapy, more conveniently. However, the results were not consistent with our expectations. There were 10 images that showed strong halos around the capsules among 72 sets of images and 14 of 47 patients (36.2%) patients showed strong halos at the thyroid bed, which might be due to the high counts. The percentage of patients with strong halos at the thyroid bed was similar to the previous report^16^. These strong halos might preclude the correct measurement of the intensity. Although we should have used geometric mean in planar images study (mean intensity of anterior and posterior images), our pilot study showed that anterior images were superior for calculating the geometric mean. The reason was not clear, but the absorption by the patient’s body or couch might have affected the intensity.

This study had some limitations. First, this was a single-center investigation with a small number of patients. However, we were able to standardize the imaging acquisition protocol in the planar and SPECT images. Second, we could not verify the results of this study. The radiation dose could be estimated based on the images, but the dose accuracy was not validated. Further research on other systems, such as the image viewers used in nuclear medicine diagnosis, is needed to validate our results. Third, there were no patients with low-risk DTC in this study; therefore, we cannot provide any recommendations for this specific patient population based on this study. Finally, we only evaluated one imaging time point. We could not evaluate ^131^I redistribution or distribution changes during RAI therapy. Multiple images must be acquired to evaluate the dynamics of the ^131^I distribution. We are planning to conduct another study to examine these distribution patterns both in time and space, calculate the absorption doses in accordance with this study, as well as determine the relationship between clinical results and absorption doses.

In conclusion, using a regression equation, we can determine the ^131^I dose in other regions, such as the thyroid bed or sites of distant metastasis. This value can be useful for the prediction of treatment results or the calculation of the actual ^131^I absorbed dose.

## Methods

### Image acquisition and analysis

We evaluated 72 sets of planar and SPECT images acquired between February 2017 and December 2018 in patients who underwent high-dose RAI therapy in our hospital. The inclusion criteria were as follows: (1) total thyroidectomy and pathologically proven DTC, (2) high-dose RAI therapy as adjuvant therapy or cancer treatment for metastases in an inpatient setting, and (3) available images for imaging analysis. The exclusion criterion was the refusal by patients to provide their images for this study. Thirty-two and 40 patients received RAI therapy as adjuvant therapy (prescribed dose was 3,700 MBq = 100 mCi) and for cancer treatment (prescribed dose was 4,810–5,550 = 130–150 mCi), respectively. The preparation of the RAI therapy was performed with thyroid hormone withdrawal (n = 39) or recombinant human thyroid-stimulating hormone (n = 43). Forty-seven patients received RAI therapy for the first time. Image acquisition was performed 3 days after ^131^I administration using a gamma camera with high-energy collimators (Infinia Hawkeye4, GE Healthcare, Milwaukee, USA). Planar images were acquired in the patient’s whole body at 15 cm/min, and the exposure time per pixel was 160 s. The matrix size was 256 × 1,024 pixels. SPECT images were acquired in a 64 × 64 matrix with step and shoot mode, and 60 projections for 10 s each. A Butterworth filter was used with critical frequency 0.32, and reconstruction was performed using the ordered subset expectation maximization (OSEM) method with 2 OSEM iterations and a max of 10 subsets. Attenuation correction with computed tomography (CT) images that were acquired at the same time and resolution recovery were performed. Scatter correction was not performed. CT images were acquired with tube potentials of 140 kV and a tube current of 2.5 mA. The slice thickness was 5 mm, and the CT rotated at 2.6 rotations per minute.

**Figure 2:**
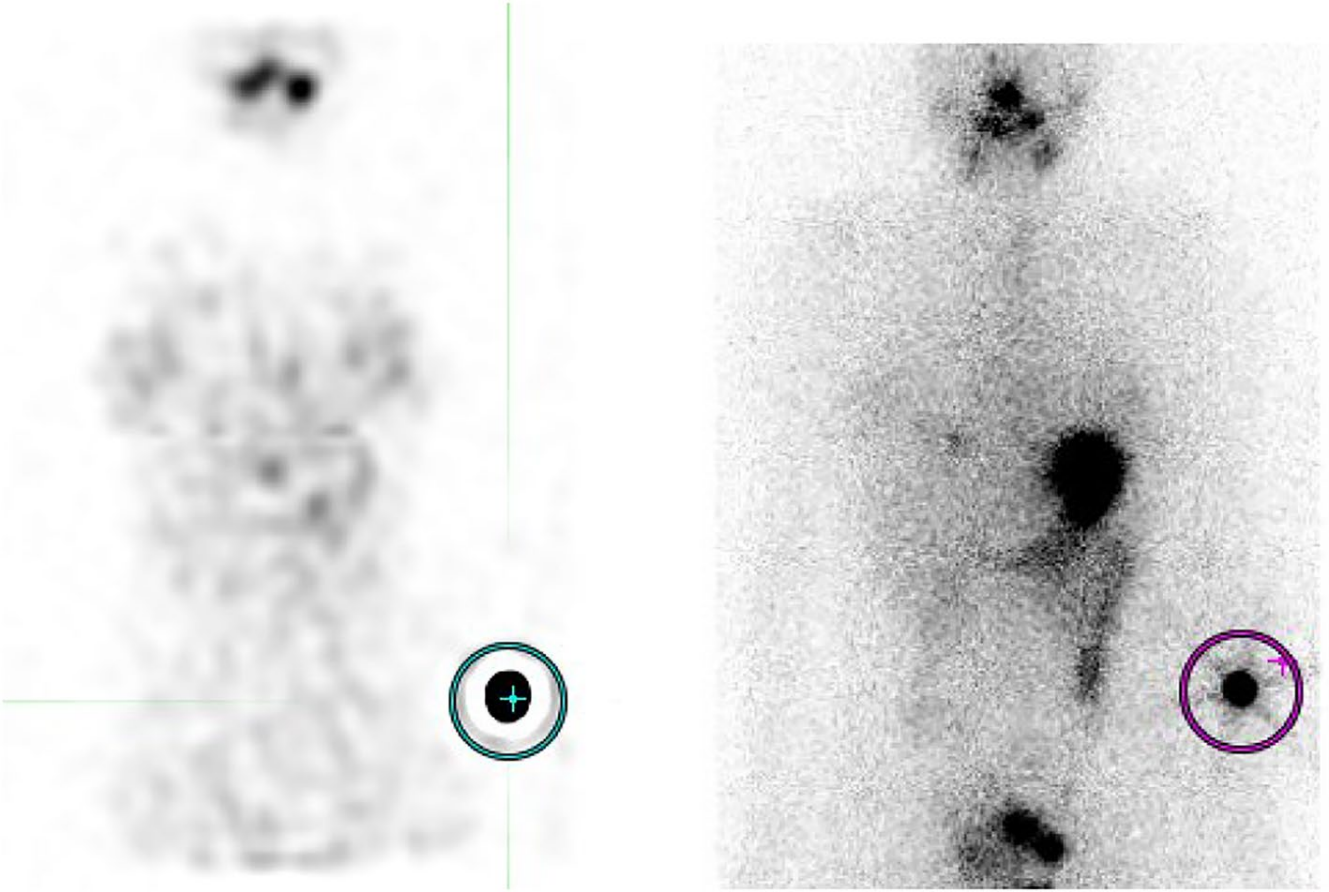
One example of the determination of region of interest (ROI). The diameter of the ROI was 5 cm and the center of the ROI was set at the center of the accumulation. Right: planar image, Left: single-photon emission computed tomography image.

We placed ^131^I capsules with their radiation doses, which were calculated based on the test date and were low enough such that it did not affect the image quality, near the patient’s arm, and planar and SPECT/CT images were acquired together with the reference. These images were transferred to a commercial treatment planning system for radiotherapy (MIM Maestro version 6.4, MIM Software, Cleveland, USA) and checked. We set the 5 cm diameter of the region of interest (ROI) on the ^131^I capsules where radiation was calculated on the day of examination and measured the intensity of the accumulation (Fig. 2). In the pilot study, we found that a 5-cm ROI performed better in evaluating the relationship between dose and intensity than ROI of other sizes. We measured the maximum and mean intensity in the ROI on planar and SPECT images and evaluated the relationship between the intensities of the ROIs and the actual radiation dose. Based on these dimensions, the ROI was planar on the planar images and spherical on the SPECT images. We tried to predict the function to estimate the radiation dose; we also estimated the radiation dose at the thyroid bed in patients who underwent RAI therapy for the first time in the same way as the reference capsule with a 5-cm ROI.

### Statistical analysis and ethical approval

All statistical analyses were performed using R software (version 3.3.1)^16^. We calculated Pearson’s correlation coefficient between the image intensity and the actual radiation dose and *p*-value. We defined a *p*-value < 0.05 as statistically significant. We used a simple linear regression model to predict the function underlying the radiation dose. We also calculated the statistical power after the evaluation. This study was performed in accordance with the 1964 Declaration of Helsinki and all subsequent revisions and followed the recommendations of the Ethical Guidelines for Medical and Health Research Involving Human Subjects. This study was approved by the Institutional Review Board (Kyoto University of Graduate School and Faculty of Medicine and Kyoto University Hospital Ethics Committee) and was performed using the opt-out method posted on our hospital website. Written informed consent was obtained from all participants of using their images for this study.

## Data Availability

The datasets generated during and/or analyzed during the current study are available from the corresponding author on reasonable request.
